# Urinalysis Using a Diaper-Based Testing Device

**DOI:** 10.3390/bios10080094

**Published:** 2020-08-10

**Authors:** Wei-Hsuan Sung, Ching-Yi Liu, Chung-Yao Yang, Cheng-Han Chen, Yu-Ting Tsao, Ching-Fen Shen, Chao-Min Cheng

**Affiliations:** 1 College of Medicine, Chang Gung University, Taoyuan 33302, Taiwan; w.h.sung0109@gmail.com; 2 Chang Gung Memorial Hospital Linkou Medical Center, Taoyuan 33305, Taiwan; cyyang@hygeiatouch.com; 3 Hygeia Touch Inc., Taipei 10441, Taiwan; itis3636@gmail.com; 4 Department of Emergency Medicine, Taipei Veterans General Hospital, Taipei 11217, Taiwan; gdc123.tw@gmail.com; 5 School of Medicine, National Yang-Ming University, Taipei 11221, Taiwan; 6 Institute of Biomedical Engineering, National Tsing Hua University, Hsinchu 30013, Taiwan; sherry450047@gmail.com; 7 Department of Pediatrics, National Cheng Kung University Hospital, College of Medicine, National Cheng Kung University, Tainan 70403, Taiwan

**Keywords:** urinary tract infection, UTI, urinalysis, diaper-based testing device, pH, leukocyte, nitrite

## Abstract

Urinary tract infections (UTI), one of the most common bacterial infections, annually affect 150 million people worldwide. Infants and the elderly are likely to have missed or delayed diagnosis of UTI due to difficulty clearly describing their symptoms. A rapid screening method for UTI is a critical and urgent need for these populations. The aim of our study is to develop a diaper-based testing device to assay urine biomarkers including pH, leukocyte, and nitrite level. This all-in-one device assists in urine collection and testing using a colorimetric approach to provide easily read visual results on the outside surface of a test strip-integrated diaper. In this study, we tested samples from 46 patients using testing strips and examined the results from 7 patients recruited to validate the strip-integrated diaper. In conclusion, this new diaper-based testing device is easy to use, rapid, and inexpensive, all of which imbue it with tremendous potential for development into a commercially viable UTI screening system.

## 1. Introduction

Diagnosis of a urinary tract infection (UTI) indicates the presence of infection anywhere in the urinary tract. Common manifestations include bacterial infections in the kidney (pyelonephritis), urethra (urethritis), and bladder (cystitis), of both genders and at a variety of ages [1]. Fever, pain in the back, burning sensation or pain when urinating, and frequent urination are common UTI symptoms [2]. UTI is one of the most common bacterial infections, annually affecting 150 million people worldwide [3]. In 2010, the direct and indirect costs of UTI in the United States amounted to approximately $2.3 billion [4]. The diagnosis of UTI depends on the execution of a urinary culture for the identification of bacterial species in the presence of clinical symptoms [5]. Consequently, UTI diagnosis, and subsequent treatment, are often missed or delayed due to the 24 to 72 h required to complete a urinary culture process. Rapid screening for urinary tract infection can reduce the costs and effort for medical treatment before it becomes more serious, especially when patients exhibit minor symptoms.

The professional urinary testing process is complicated for most people to accomplish. On the other hand, for those who may not have specific symptoms or who may have trouble describing their symptoms clearly, like infants, children, and the elderly, a rapid and easily operated urinary screening method is an urgent need [6]. The introduction of point-of-care testing (POCT) allows for more timely diagnosis and rapid clinical decisions toward targeted treatment [7]. Urine dipsticks are a prominently used example of a commercially produced POCT tool for rapid urinary infection testing [8]. There is, however, still room to consider and develop a urine-sampling POCT device that is less costly and more user-friendly. Several groups have developed the concept of a cotton-based smart diaper. Cotton, a natural material, provides excellent flexibility and biocompatibility for detection devices. Lin et al. have underscored the particular advantages of a cotton-based testing device by accurately obtaining semi-quantitative information in a buffer system [9]. Seo et al. presented a diaper-embedded and self-powered UTI monitoring sensor module [10]. Couto et al. described an eight-layer microfluidic paper-based device for screening and analysis of multiple biomarkers from urine samples on diapers [11,12].

Above all, there has been an urgent need to integrate urine sampling, testing, and analysis to build a convenient UTI screening solution that would benefit specific, at-risk populations, especially those who are have difficulty clearly describing their symptoms. We felt that novel advances could be made to improve the efficiency of UTI screening by using point-of-care testing (POCT) techniques that simultaneously integrated urine collection, screening, and analysis. In this study, we aimed to develop an accurate, inexpensive, rapid, and easy to use in-vitro diagnostic device (IVD) to detect UTIs in infants and the elderly. Leveraging the characteristics of cotton and using specific biological indicators for bacterial infection, i.e., pH value, leukocyte, and nitrite concentrations in urine, we have developed a diaper-based testing device that integrates sampling and UTI detection in a single, convenient package.

## 2. Materials and Methods

### 2.1. Screening Reagents for Urinary Tract Infections (UTI) Detection

For UTI biomarker selection and for screening reagent formulation, we used Hygeia Touch Urinary Tract Infection Test Strips (US FDA listing, Hygeia Touch Inc., Hsinchu, Taiwan). It has been suggested that the use of POCT urine analyzers would increase the quality of urinalysis. With the introduction of commercially available POCT urine analyzers, it may be possible to improve the quality of urinalysis in general practice, but such analyzers must demonstrate good analytical performance and agreement with laboratory standards, and data to support these qualities is still lacking. The Hygeia brand test strips can be used as a quick self-screening aid to detect UTI, for people with symptoms such as fever, back pain, uncomfortable burning or pain upon urinating, and frequent urination. The test strips simultaneously identify three UTI indicators: urinary pH value, leukocyte level, and nitrite level. The screening reagents for each indicator are coated on independent test pads, and act as the reaction zone of each strip.

The screening reagents were formulated for a colorimetric assay to provide unique UTI-associated gradient color changes associated with UTI infection. When the urine being tested contains bacteria, these reagents change in color from light orange to dark green to indicate a high pH value, from light yellow to pink or purple to indicate the abnormal presence of leukocytes, and from light yellow to pink to indicate a high nitrite level. The colorimetric reaction of each reagent is based on chemical reactions with specific components in urine. The leukocyte testing reagent, i.e., 0.5% *w/w* pyrrole amino acid ester (CAS No. 100750-39-8, Santa Cruz Biotechnology, Dallas, TX, USA) + 0.4% *w/w* diazonium salt (CAS No. 101-89-3, MilliporeSigma, St. Louis, MO, USA) detects leukocyte esterase hydrolysis of ester derivatives, the nitrite testing reagent detects the formation of diazonium compounds based on the reaction of nitrite and 4.5% *w/w* p-arsanilic acid (CAS No. 98-50-0, MilliporeSigma, St. Louis, MO, USA), and the pH testing reagent uses dual indicators, i.e., 0.5% *w/w* methyl red sodium salt (CAS No. 845-10-3, MilliporeSigma, St. Louis, MO, USA) + 5% *w/w* bromthymol blue (CAS No. 76-59-5, Tokyo Chemical Industry, Tokyo, Japan). We immersed the test strip into reagents for 5 min and then allowed it to dry for 24 h.

### 2.2. Clinical Evaluation for UTI Test Strip

**Figure 1:**
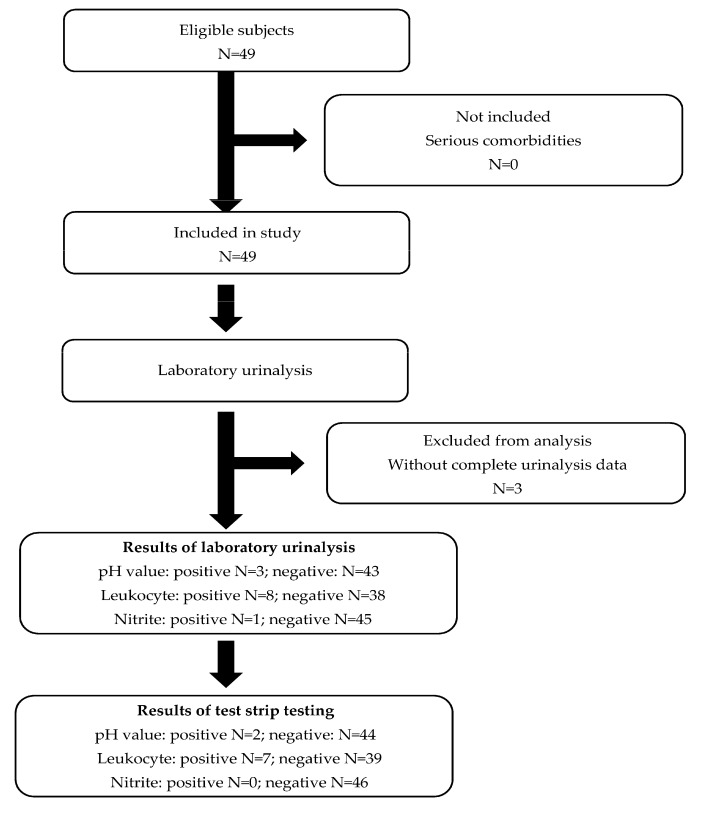
An overview of the clinical evaluation and process flow. Urine samples were provided by all subjects for comparative analysis. Samples were evaluated using typical laboratory urinalysis and test strips, and results from both methods were compared.

A comparison test was performed to evaluate screening reagent capability. A clinical evaluation (N = 49) of Hygeia Touch Urinary Tract Infection Test Strips was used to evaluate the accuracy of the testing reagents, which were then integrated into our diaper-based testing device. The urine test strip detection results were compared to the laboratory analysis from Artemis Hospital (Kaohsiung, Taiwan) as our laboratory reference standard. UTI diagnosis is based on the detection of pathogens in the presence of clinical symptoms. However, laboratory urinalysis, not clinical diagnosis, was selected as the reference standard in this study because clinical diagnosis of UTI is not necessarily related to the presence of nitrite, leukocyte, or high alkalinity level in urine. The standard was calibrated prior to executing the study. All urine testing and results reading procedures were performed at Artemis Hospital (Kaohsiung, Taiwan). The study flowchart is presented in Figure 1. Forty-nine urine samples were collected from patients who delivered a urine sample for a routine investigation. Women above 18 years old were included, and those with severe comorbidities were excluded. Each subject was enrolled only once. Three subjects without complete urine analysis data were excluded from analysis.

### 2.3. Diaper-Based Testing Device Fabrication

**Figure 2:**
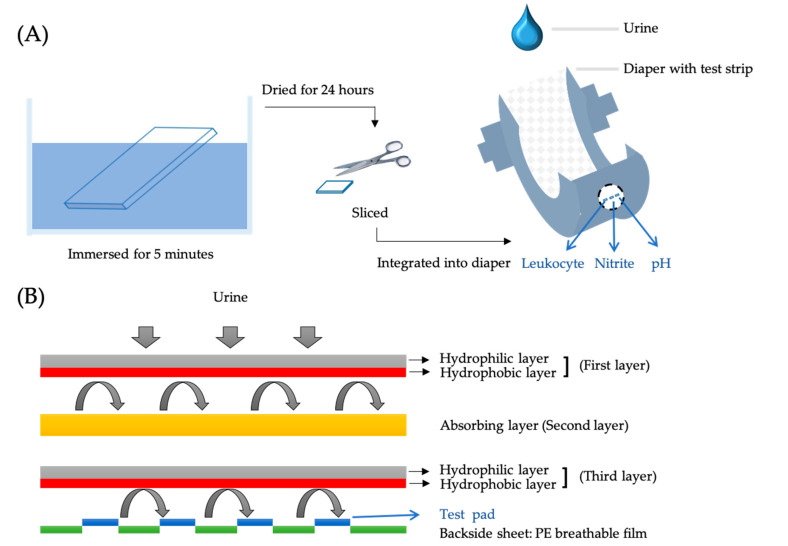
(**A**) The fabrication process for our diaper-based testing device. The test strip was immersed into various diagnostic reagents for 5 min and dried for 24 h at room temperature. The test strip was integrated into a diaper, attached to the breathable plastic backside sheet, allowed the results to be visible from the outside. (**B**) Schematic of the structure of our diaper-based testing device. The test strip was integrated between the third layer and backside sheet, which allowed the colorimetric result to be visible from the outside.

The diaper-based testing device was designed to provide multiple biomarker detection values with a focus on accuracy, ease-of-use, and cost-effectiveness. A schematic of the fabrication process is provided in Figure 2A. The diaper-based testing device comprised two primary parts: (1) a test strip (Hygeia Touch Inc., Hsinchu, Taiwan); and, (2) a multi-layered cotton diaper with an outer layer of plastic substrate [9]. As shown in Figure 2B, urine was deposited onto the first layer of cotton. The function of the first layer was to pass the liquid to the subsequent absorbent layer. The reverse side of this layer was hydrophobic and prevented urine backflow toward the skin. The function of the second layer was to absorb the liquid sample and deliver it to the third layer. The third layer was constructed of the same type of cotton as the first layer—it absorbed urine, passed urine to the outer hydrophobic side, and prevented backflow toward the second layer. The biomarker-impregnated test strips were implanted between this layer and the final plastic back layer, which was designed to keep liquid inside without leaving the diaper. We leveraged these material characteristics to create effective, one-directional flow for urine sample delivery, allowing fluid to pass to the target test strips while removing impurities. As samples reached the test strips, a colorimetric reaction ensued. The entire procedure, from sample to test, was completed in a single step. This facilitated urine sampling and testing in real time.

### 2.4. Statistics Analysis

We analyzed, evaluated, and compared data collected from test strips and regular laboratory urinalysis method from Artemis Hospital. To compare UTI test strip performance regarding pH value, leukocyte level, and nitrite level with the laboratory reference standard, we used contingency (2 × 2) tables to calculate sensitivity, specificity, agreement, positive predictive value (PPV), negative predictive value (NPV). Sensitivity referred to how often the test was positive when the result of the laboratory reference standard was positive, while specificity indicated how often the test was negative when the result of the laboratory reference standard was negative. The overall agreement was defined as the consistent probability between the test strip results and the laboratory reference standard results. The positive predictive value (PPV) was defined as the probability that a positive result truly indicated the presence of infection. The negative predictive value (NPV) was the probability that a normal result indicated the patient did not have an infection. Statistical analyses were performed using SPSS computer software (SPSS Inc., Chicago, IL, USA). 

## 3. Results

To evaluate the accuracy of the three UTI detection reagents used in this study, we collaborated with Artemis Hospital to employ Hygeia Touch Urinary Tract Infection Test Strips for preliminary screening. We enrolled patients for eligibility over a two-month period from March 1st, 2018 to April 30th, 2018. During this time, 49 participates agreed to join our study. An overview of subject inclusion and exclusion details is provided in Figure 1. The age of subjects ranged from 22 to 40 years (30.3 ± 4.9 years), and all of them were female. Forty-nine anonymous urine samples from subjects were collected and analyzed. Samples were first analyzed using laboratory-standard methods in Artemis Hospital and then analyzed with urine test strips. Both processes were performed by a well-trained, registered nurse, who was responsible for test strip operation, colorimetric result reading, and data recording. All testing was anonymized.

**Figure 3:**
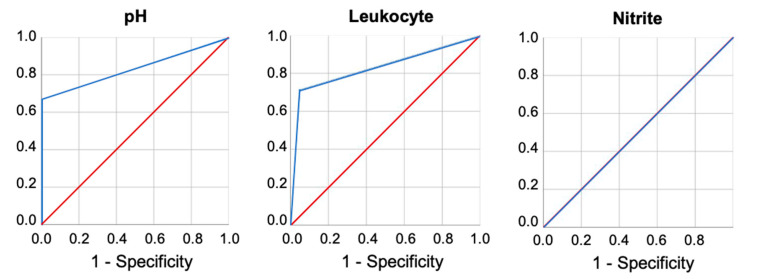
Receiver operating characteristic (ROC) curves of pH, leukocyte, and nitrite. The realization of the receiver operating characteristic (ROC) curve found the area under the curve (AUC) of 0.833 in pH, 0.832 in leukocyte and 0.5 in nitrite.

There were no subjects with severe comorbidities. Therefore, all 49 subjects were enrolled in this study. From the results of laboratory urinalysis, three samples did not have complete nitrite test results, and were excluded from data analysis. The laboratory results indicated that four samples were alkaline (pH > 8, indicating bacterial infection), nine samples were leukocyte positive (>5 leukocytes per high power field), and one sample was nitrite positive. From the test strip results, two samples had positive pH values, seven samples were leukocyte positive, and none were nitrite-positive samples. All of the included data were analyzed and are statistically summarized in Table 1. Data includes sensitivity, specificity, PPVs, and NPVs, as well as the agreement for pH, leukocyte, and nitrite values for both the test strip and the reference-standard laboratory urinalysis. By plotting the pairs of sensitivity and corresponding specificity, we obtained a receiver operating characteristic (ROC) curve (Figure 3). Clinical validation of the test strip showed that, for pH value, the specificity, agreement, PPV, and NPV were high (100%, 97.83%, 100%, 97.73%), despite the sensitivity (66.67%). Leukocyte detection showed a similar status, with the specificity, agreement, PPV, NPV of 94.74%, 89.13%, 71.43%, 92.31%, but lower sensitivity (62.5%). For the nitrite test, the specificity, agreement, and NPV showed positive results for the nitrite detection reagent (100%, 97.83%, 97.83%). We were unable to calculate sensitivity and PPV because the sample number for nitrite-positive urine was less for both the laboratory standard and the test strip. As a result, sensitivity and PPV were not applicable for the nitrite test. Since our inclusion criteria did not specifically include patients with UTI, values for pH, leukocyte-, or nitrite-positive urine sample size was too small to reflect sensitivity in both the laboratory urinalysis and the UTI test strip testing. This is because the sensitivity and PPV decrease as prevalence decreases. For a mathematical explanation of this phenomenon, we can use PPV as an example. PPV is defined as (sensitivity × prevalence)/[(sensitivity × prevalence) + ((1 − specificity) × (1 − prevalence))]. Therefore, as prevalence decreases toward zero, the value of (sensitivity × prevalence) goes towards zero, driving the whole denominator to smaller and smaller values as prevalence decreases. That is to say, if our series had a higher prevalence of UTI, there would be an increase in PPV along with a decrease of NPV. However, our current study demonstrates that when the test strips generated positive results for high pH or leukocytes, urinalysis results showed similar results. These results indicate the applicable potential for clinical adoption of these UTI detection reagents as a tool for UTI screening. This confirms that the currently tested POCT urine analyzer performed sufficiently well for use in primary care practices.

After evaluating the results from using the UTI detection reagents coated on the UTI test strip, we further developed the diaper-based testing device. The sample volume used was 10 mL. This protocol has been approved by Institutional Review Board of Taipei Veterans General Hospital (IRB 2019-10-001B approved 25 October 2019). The test strips were coated with detection reagents separately and then integrated into the diaper, as shown in Figure 2A. To prevent the reagent from coming into direct contact with human skin, and to provide a better result reading process, the test pad (of the test strip) was embedded into a diaper. The outer surface of the test pad was visible on the outer surface of the diaper. This design allowed for efficient and safe sample collection and readily observable colorimetric results.

**Figure 4:**
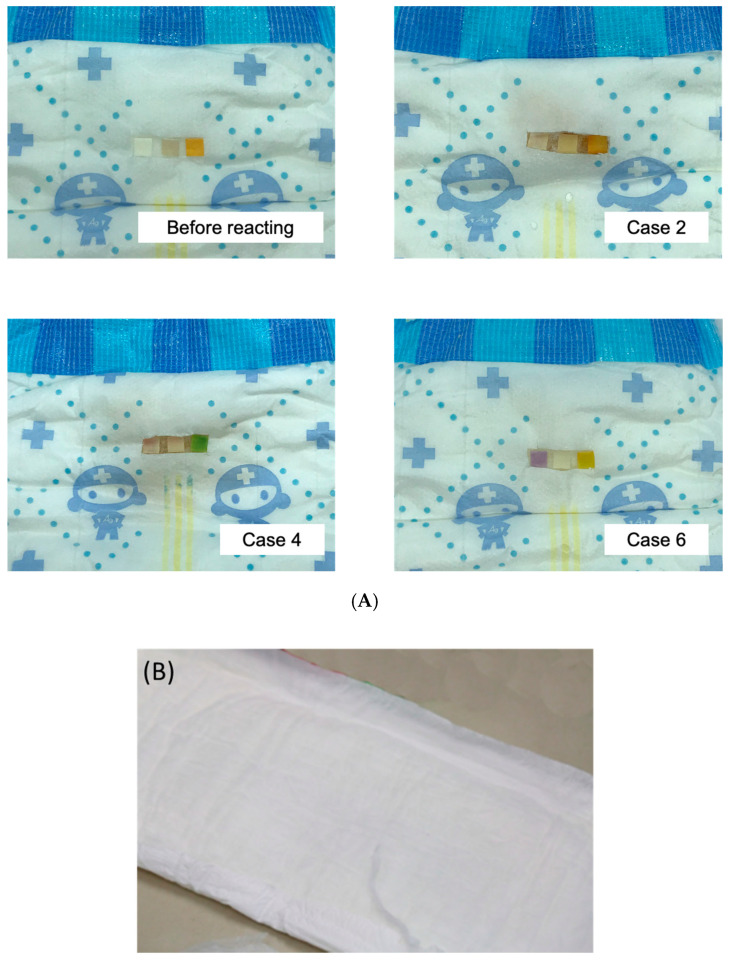
The representative images of the diaper-based testing device prototype. (**A**) Test strips were coated with indicated reagents to detect urinary leukocyte, nitrite, and pH levels (from left to right). The colorimetric results from case 2 showed no color changes. The colorimetric results from case 4 showed positive results for the detection of leukocytes and nitrite. The colorimetric results from case 6 showed positive results for the detection of leukocytes, and no other color changes. (**B**) After reacting for 1 h, no backflow appeared on the inside of the used diaper.

Figure 2B is a schematic of the diaper-based testing device construction. In this device, urine contacts the first, initial layer of the diaper and is absorbed so that it flows from one side of that layer to the other side and then to the second layer. The side of the first layer that is away from the body has hydrophobic properties that inhibit urine flow back toward the body. Urine is absorbed into the second absorption layer and then passed to the third layer. The test strip is embedded on the other side of this third layer, so that it absorbs the urine sample. The diaper-based testing device prototype is shown in Figure 4. We fabricated the diaper-based testing device prototype as shown in Figure 4A. The left picture shows the device post-production and demonstrates how the test strip was adhered to the back of the diaper under a transparent layer of plastic, which allows the test pad (of the test strip) to be visible from the outside. Test strips were coated with indicated reagents to detect urinary leukocyte, nitrite, and pH levels (from left to right). Test strips were integrated into the back of the diaper and under a transparent plastic film so that results were visible on the outside of the device. The colorimetric results from case 2 showed no color changes; the device did not detect leukocytes, nitrite, or a pH value of 6 or greater. The colorimetric results from case 4 showed color changes from light yellow to light pink (indicating leukocyte detection), from light yellow to pink (indicating nitrite detection), and from light orange to dark green (indicating the detection of a pH value of 7). The colorimetric results from case 6 showed color changes from light yellow to purple (indicating leukocyte detection), and no other color changes that would indicate the presence of nitrite or a pH value of 6 or greater. After waiting for an hour, we also observed the inside surface of the diaper, to evaluate whether there was any backflow; in Figure 4B, images from inside the diaper show the clean surface, which indicates that the diaper displayed great absorption and prevented backflow. In Table 2, we provide patient demographics to include gender, age symptoms, underlying diseases, results of urinalysis and diaper-based testing device. These results indicate that our test-strip-integrated diaper is suitable for detecting the presence of urine constituents such as nitrite and leukocytes. The embedded test strip design facilitated outside visibility, a user-friendly feature, and did not affect colorimetric reaction efficiency.

The visually recognizable colorimetric results provided by our testing device support its potential for laboratory-based studies, clinical use, and personal use at home. By embedding UTI detection reagents into test strips that were then integrated into a diaper, we created a diaper-based testing device prototype. The colorimetric method provides clear results for caregivers to observe possible UTI, which is especially helpful for individuals who face difficulties expressing symptoms, i.e., infants, children, and the elderly.

## 4. Discussion

In this study, it was shown that the UTI screening reagents could be applied in an interesting way to screen for UTI in a variety of populations and across age groups, especially in infants, children, and the elderly. These populations are at high risk of UTI due to consistent diaper wearing, and they may face difficulties expressing symptoms of infection. UTI diagnosis is often missed during physical examinations due to the non-specific symptoms and signs, such as fever, dysuria, frequency, urgency, suprapubic pain, or hematuria in these age groups [13]. It would be particularly useful for them to have a rapid, early-stage, user-friendly approach for UTI screening. For this reason, we created a device that integrated urine collection, biomarker-based screening, and results reading.

Screening and early diagnosis of UTI during infancy is highly recommend, since Infantile UTI possibly is associated with urinary tract malformations and therefore contributes to renal scarring and consecutive chronic kidney disease (CKD) [14]. Notably, integrating the screening reagents into a diaper will help obtain and process urine samples quickly and easily, reduce the risk of using invasive sampling methods, such as transurethral bladder catheterization or suprapubic aspiration. The highest prevalence of UTI is actually in the overall elderly population.

UTI strongly influences not only community-dwelling elderly but also highly functionally impaired long-term care facility residents [15]. The diagnosis of symptomatic UTI in older adults with associated disorders such as Alzheimer’s disease, Parkinson’s disease, and stroke are extremely challenging [16]. Elderly patients suffering from functional or cognitive impairments have great difficulty communicating localized genitourinary symptoms [2]. Our testing devices may help urine sample collection and bacteriuria screening in these populations [17]. The design of the testing device promoted urine flow to our test strips and facilitated results observation from the outside, which provided considerable convenience for users and caregivers.

Normal urine pH is slightly acidic, ranging from 4.5 to 8.0. A urine pH of 8.5 or 9.0 is indicative of urea-splitting organisms [18]. In a previous study, a portable electronic pH meter was found to provide more accurate results than commonly used dipsticks or test strips [19]. In comparison, our UTI testing reagents were based on colorimetric analysis and demonstrated considerable specificity, agreement, PPV, and NPV, without electrically powered or complicated machine readers. This screening method might be more convenient for general users to read results and perform urinary screening.

For the detection of leukocytes, we compared the analytical performance of our test strip with reagents to other commercially available POCT urine analyzers (see Table 3) [20]. Our test strip demonstrated comparable specificity, NPV, and agreement. The sensitivity and PPV of our test strip were lower than those for other POCT urine analyzers, but this may be due to the fact that few UTI-positive subjects enrolled in our study.

For nitrite, our test strip has comparable specificity, agreement, and NPV compared to the reference method [20]. However, the sensitivity and PPV were not applicable for our nitrite tests due to the limited number of nitrite-positive urine samples. The sensitivity of the nitrite-detecting reagent requires more data for better analysis—we believe that increasing the number of study subjects would provide a more convincing result.

There are some limitations to our study. First, we included only a small number of patients, i.e., 46 patients for testing strips (note that only 3, 8, and 1 patients displayed positive results for the detection of pH, leukocyte, and nitrite, respectively, using laboratory urinalysis), and 7 patients for the strip-integrated diaper, which makes it difficult to draw strong conclusions. Second, our approach itself is the difficulty isolating and capturing midstream samples, which would be more ideal for urine test accuracy. Third, although our diaper-based testing device was sterilized following production and both sampling and biomarker detection were executed in a single step, there is still a chance of contamination due to collection under non-sterile conditions. The test strip test area remains stable for two years. The test strips on our diaper-based testing device prototype are stable for at least one month, a timespan that will be improved to two years following additional engineering efforts. Fourth, just like most of other urinary POCT device, our testing device detects a possible UTI, but does not identify the precise casual pathogenic microorganism. Although we have evaluated the UTI detecting reagents, clinical validation of our diaper-integrated test strip requires more clinical evidence to improve the sensitivity and specificity.

Overall, in comparison to conventional detection methods, our diaper-based testing device prototype features many advantages: (1) it detects multiple clinical targets in a single step using one device, which provides better user-friendliness; and, (2) it is inexpensive and can be used as a POCT device with little training or equipment. The novelty of the design is primarily derived from the capacity for simultaneous detection of urine pH, nitrite, and leukocyte levels combined with the complexity for collecting and testing using a single, diaper-based POCT device. Further, we expect this particular diaper-based testing device technology to translate well into the development of a commercial POCT device correlated due to the high degree of technical capability. Notably, this device may offer significant advantages for screening in a variety of areas, including rural/remote areas and long-term healthcare centers.

## 5. Conclusions

In this study, we demonstrated a diaper-based testing device that is inexpensive, portable, and easy to use. UTI-screening diapers integrated with biomarker-impregnated test strips would offer great potential for use with infants, children, and the elderly. This methodology may be used at home and in short- and long-term healthcare settings and offers promise for convenient UTI screening of individuals in remote and resource-poor areas.

## Figures and Tables

**Table 1:** The clinical evaluation summary of urinary tract infections (UTI) detection reagents. Comparisons of pH value, nitrite, and leukocyte concentration between the test strip and clinical laboratory urinalysis are shown. Sensitivity, specificity, agreement, positive predictive value (PPV), and negative predictive value (NPV) were analyzed in this study. (PPV, positive predicted value. NPV, negative predictive value. N, the sample number in each condition.).

**pH Value**				
		Laboratory urinalysis (N)		
		Positive	Negative	Total
Test strip (N)	Positive	2	0	2
	Negative	1	43	44
	Total	3	43	46
**Validity**	**Specificity**	**Agreement**	**PPV**	**NPV**
66.67%	100.00%	97.83%	100.00%	97.73%
**Leukocyte Level**				
		Laboratory urinalysis (N)		
		Positive	Negative	Total
Test strip (N)	Positive	5	2	7
	Negative	3	36	39
	Total	8	38	46
**Validity**	**Specificity**	**Agreement**	**PPV**	**NPV**
62.50%	94.74%	89.13%	71.43%	92.31%
**Nitrite Level**				
		Laboratory urinalysis (N)		
		Positive	Negative	Total
Test strip (N)	Positive	0	0	0
	Negative	1	45	46
	Total	1	45	46
**Validity**	**Specificity**	**Agreement**	**PPV**	**NPV**
N/A	100.00%	97.83%	N/A	97.83%

**Table 2:** The demographics of patients enrolled in the diaper-based testing device including gender, age symptoms, underlying diseases, results of urinalysis, and diaper-based testing device results.

	Gender	Age	Symptoms				Underlying Diseases			Urinalysis			Diaper-Based Testing Device		
			Shaking Chills	Dysuria	Increased Urinary Frequency	Flank Pain	DM	Uremia	CKD	pH	Leukocyte	Nitrite	pH	Leukocyte	Nitrite
**Case 1**	F	84	N	N	N	N	P	P	P	6	N	N	5	N	N
**Case 2**	F	72	N	N	N	N	N	P	P	6	N	N	6	N	N
**Case 3**	M	88	N	N	N	N	N	N	N	7	N	N	5	N	N
**Case 4**	M	67	N	N	N	N	P	N	N	6.5	P	P	7	P	P
**Case 5**	M	66	N	N	N	N	N	N	N	5.5	N	N	6	N	N
**Case 6**	F	57	P	P	P	P	N	N	N	6	P	N	6	P	N
**Case 7**	F	69	N	N	N	N	N	N	N	5	N	N	5	N	N

F: female; M: male; N: negative; P: positive; DM: Diabetes Mellitus; CKD: Chronic Kidney Disease.

**Table 3:** Comparisons of leukocyte detection capability of test strips. Comparison of the sensitivity, specificity, PPV, NPV, and agreement for the leukocyte reagent used in this study, and other point-of-care testing (POCT) urine analyzers in the detection of leukocytes.

	Sensitivity (Validity)	Specificity	PPV	NPV	Agreement
Hygeia Touch Test Strip	62.50%	94.74%	71.43%	92.31%	89.13%
Clinitek Status (Siemens)	56%	100%	100%	76%	60.04%
Uryxxon Relax (Macherey Nagel)	94%	98%	97%	96%	91.94%
Urisys 1100 (Roche)	78%	100%	100%	87%	80.67%
Aution micro (Menarini)	94%	98%	97%	96%	91.94%
Aution 11 (Menarini)	93%	96%	94%	96%	89.31%
Urilyzer (Analyticon)	66%	100%	100%	80%	69.05%
